# No effect of delay on the spatial representation of serial reach targets

**DOI:** 10.1007/s00221-015-4197-9

**Published:** 2015-01-20

**Authors:** Immo Schütz, Denise Y. P. Henriques, Katja Fiehler

**Affiliations:** 1 Department of Psychology, Justus-Liebig-University Giessen, Otto-Behaghel-Str. 10F, 35394 Giessen, Germany; 2 Center for Vision Research, School of Kinesiology and Health Science, York University, 4700 Keele Street, Toronto, ON M3J 1P3 Canada

**Keywords:** Gaze-centered spatial updating, Memory delay, Reaching, Egocentric, Allocentric, Reference frame

## Abstract

When reaching for remembered target locations, it has been argued that the brain primarily relies on egocentric metrics and especially target position relative to gaze when reaches are immediate, but that the visuo-motor system relies stronger on allocentric (i.e., object-centered) metrics when a reach is delayed. However, previous reports from our group have shown that reaches to single remembered targets are represented relative to gaze, even when static visual landmarks are available and reaches are delayed by up to 12 s. Based on previous findings which showed a stronger contribution of allocentric coding in serial reach planning, the present study aimed to determine whether delay influences the use of a gaze-dependent reference frame when reaching to two remembered targets in a sequence after a delay of 0, 5 or 12 s. Gaze was varied relative to the first and second target and shifted away from the target before each reach. We found that participants used egocentric and allocentric reference frames in combination with a stronger reliance on allocentric information regardless of whether reaches were executed immediately or after a delay. Our results suggest that the relative contributions of egocentric and allocentric reference frames for spatial coding and updating of sequential reach targets do not change with a memory delay between target presentation and reaching.

## Introduction

Goal-directed reaching movements are an essential part of human interactions with the world. Whether we want to reach out for a coffee cup, push a button or interact with a touch-enabled device, in all cases, our brain needs to store and represent the spatial location of a movement goal and use this representation when controlling movement. In order to encode spatial locations for action, the brain can utilize multiple frames of reference, either separately or in combination (Colby 1998; Soechting and Flanders 1992). These reference frames are typically divided into two main classes: egocentric reference frames, which code target locations relative to the observer’s position and posture such as current eye, head, gaze or hand position, and allocentric reference frames, which code targets relative to other external landmarks or stimuli (Colby 1998).

Behavioral studies in humans suggest that targets for goal-directed reaching are predominantly coded in a gaze-centered egocentric frame of reference, at least when reaching to a single target in the dark without visual feedback of the hand (Henriques et al. 1998; Crawford et al. 2011). Gaze-dependent representations have been found for immediate (Beurze et al. 2006; Henriques et al. 1998; Medendorp and Crawford 2002) as well as delayed reaching (Fiehler et al. 2011; Khan et al. 2005a, b, Schütz et al. 2013). Typically, these reaching studies find systematic reach endpoint errors: When reaching to remembered targets, subjects overshoot the target opposite of current gaze direction (Bock 1986), even when the target was previously foveated and gaze was then shifted into the periphery (Henriques et al. 1998). With regard to the neural basis of these representations, electrophysiological results in non-human primates suggest the posterior parietal cortex (PPC) as a key structure for sensorimotor integration, which represents spatial goals in a gaze-centered frame of reference (Andersen and Buneo 2002; Batista et al. 1999, Colby and Goldberg 1999) and updates these locations with every eye movement (Duhamel et al. 1992). Human neuroimaging studies have proposed many homologs between monkey and human parietal areas relevant for reach control (Culham et al. 2006; Filimon 2010; Grefkes and Fink 2005) and also found evidence for gaze-centered spatial updating of reach targets in the human PPC (Medendorp et al. 2003; Medendorp et al. 2005; Beurze et al. 2010).

In addition to egocentric information, the brain can also utilize allocentric information when encoding spatial locations, such as landmarks or a structured background (Carrozzo et al. 2002; Krigolson and Heath 2004). For example, visual landmarks during encoding of a reach goal have been shown to increase both accuracy and precision of reach movements (Hay and Redon 2006; Krigolson and Heath 2004; Krigolson et al. 2007; Obhi and Goodale 2005). Recent behavioral studies found that when visual landmarks were available, gaze-dependent reach errors were reduced but still present, indicating a combined use of egocentric and allocentric visual information (Byrne et al. 2010; Schütz et al. 2013). This is in line with results showing that egocentric and allocentric information are integrated in a statistically optimal fashion during reach planning (Byrne and Crawford 2010; McGuire and Sabes 2009); however, in some cases, one representation seems to be selected over the other depending on task and stimulus properties (Byrne and Henriques 2012).

The temporal characteristics of a motor act are supposed to influence the frame of reference. Immediate reaching is suggested to rely on an egocentric frame of reference (Westwood and Goodale 2003), and such egocentric information has been shown to quickly decay after target offset (Chen et al. 2011; Hesse and Franz 2009, 2010). Executing movements after even a short delay, however, is assumed to make use of a perception-based allocentric representation (Goodale and Milner 1992; Hu and Goodale 2000; Westwood et al. 2000; Westwood and Goodale 2003), which can persist for longer periods of time without degradation (Chen et al. 2011; Hay and Redon 2006). In memory-guided movement tasks where both egocentric and allocentric information are available, allocentric coding has also been reported to take precedence (Lemay et al. 2004; Sheth and Shimojo 2004).

Based on these findings, a switch from egocentric to allocentric coding would be expected when a reach movement is delayed compared with immediate movement execution (Westwood et al. 2003). However, at least in a delayed reaching task from our laboratory that was devoid of any other visual information, delay had no effect on gaze-dependent reach errors (Fiehler et al. 2011). When static visual landmarks were added to the display in a later study, the amount of delay between target viewing and reaching (0, 8 or 12 s) still had no influence on the pattern of reach errors, although overall errors were reduced when the landmarks were available (Schütz et al. 2013). These results suggest a dominant contribution of a gaze-dependent egocentric representation for immediate and delayed reaching.

Besides single-target reaching tasks, the relative contributions of egocentric and allocentric visual information have also been investigated in reaching to multiple visual targets. In a previous study by Thompson and Henriques (2010), subjects sequentially fixated two visual targets which were always separated by a constant distance of 5°. Therefore, the second target could be represented using its fixed distance relative to the first target when coding and launching a subsequent reach to the second target. After target presentation, they shifted gaze to a fixation position and reached to the first remembered target, then shifted gaze again and reached to the second remembered target location. Subjects did not return the reaching hand to the start position between the first and second reaches; rather, they held their index finger in place after the first reach and while shifting gaze to the second fixation LED, and then reached from the first to the second remembered target position. If participants coded the second target solely based on the constant distance between the targets, these reaches should have amplitudes close to 5°. On the other hand, if they used only egocentric gaze information to code the second target’s location, reach amplitudes should vary systematically with gaze as described above for the single-target paradigm. It was found that subjects’ actual reach amplitudes fell in between both predictions, suggesting a combined use of egocentric and allocentric information when coding the second target location (Thompson and Henriques 2010).

Due to the fact that subjects executed a reach from the first to the second target instead of performing two separate goal-directed movements, the first target not only constituted a possible visual cue when coding the second target location, but was also a reliable intermediate reach goal for the second reach, forcing subjects to compute the constant relative distance between both targets in order to correctly execute the second reach. This is in contrast to our earlier landmark study (Schütz et al. 2013), where the landmarks only provided a visual boundary of the workspace, but did not serve as a possible reach goal by themselves. It is therefore conceivable that subjects in the Schütz et al. (2013) study did not use the landmarks to improve reach accuracy with delay because they were not relevant for the task at hand. This assumption is supported by recent work from our group demonstrating that landmarks can influence reach endpoints, but only when they were potential reach targets and therefore relevant for the subjects’ task (Fiehler et al. 2014; Klinghammer et al. 2014).

**Fig. 1:**
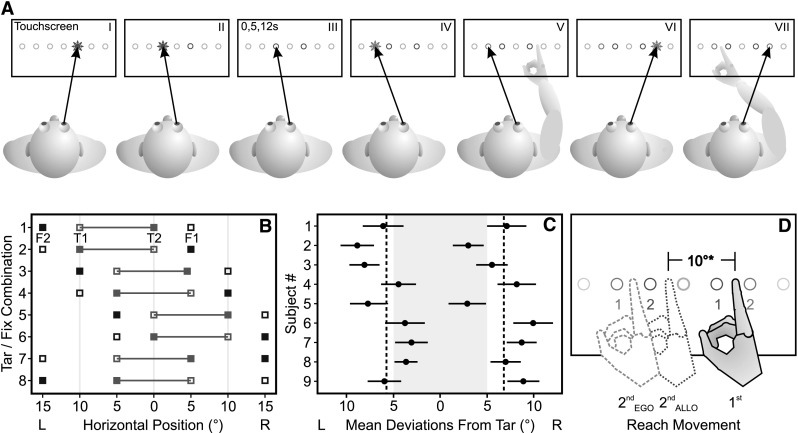
Methods. **a** Experimental paradigm. Subjects sequentially fixated two red LED targets (*I*, *II*) always spaced 10° apart. After a delay of 0, 5 or 12 s (*III*), during which they maintained gaze at the second target location without visual feedback, a green fixation LED was lit. Subjects shifted their gaze to this location (*IV*) and when it turned off, reached for the first target (*V*). They then shifted gaze to a second fixation LED (*VI*) before they reached for the second target (*VII*). **b** Target (connected *red squares*) and fixation positions (separate *green squares*) used in the experiment. *Open symbols* indicate the first stimulus in the sequence, *filled symbols* the second. Targets were always spaced 10° apart. **c** Individual fixed inter-target distance biases. Data points denote mean *left* and *right* touches for each subject. *Error bars* show ±1 standard error. *Gray*-shaded area indicates actual target positions. *Dashed lines* illustrate mean distance bias (12.7° ± 1.5°). **d** Predictions for second reach. *Filled hand* indicates first reach endpoint shifted to the right of the target (*red* 1). *Blue*, *dotted hand* illustrates allocentric prediction for the second reach based on the subjective fixed inter-target distance (10°*). *Orange, dashed hand* illustrates the egocentric gaze-centered prediction based on the first reach. *Red circles* indicate first (1) and second (2) targets. *Green circles* likewise represent first (1) and second (2) fixation LEDs (color figure online)

The present study investigated the influence of a memory delay on the use of egocentric gaze-centered and allocentric reference frames in serial reach planning. To test this, we used a two-target sequential-reaching task adapted from Thompson and Henriques (2010) and varied the delay between target presentation and reach. Based on previous studies (Schütz et al. 2013; Thompson and Henriques 2010), we predict egocentric, gaze-dependent coding and updating of the first reach target because no allocentric reference is available. For the second reach, two possible outcomes are hypothesized which are illustrated in Fig. 1d. If the second target is represented in an egocentric, gaze-dependent reference frame, we expect reach endpoints to vary systematically with gaze (orange dashed hand), analogous to the results of the first reach. If the second reach target is coded allocentrically using its constant distance relative to the first target, we expect reach endpoints about 10° apart from the first reach endpoint (blue dotted hand). Thus, reach amplitudes (distances of reaches from the first to the second target) predicted by an egocentric, gaze-centered reference frame should fluctuate systematically and substantially, while reach amplitudes predicted by allocentric information should be constant. If the relative contribution of allocentric information increases at longer delays, we expect a greater influence of the constant allocentric distance between the targets on the endpoints of the second reach when movements are delayed. Movement amplitudes of the second reach should approach the constant 10° inter-target distance with increasing delay, and systematic variation between reach amplitudes at different gaze deviations should be small in delayed reaches.

## Materials and methods

### Participants

Ten naive volunteers participated in this experiment. One subject was removed from final analyses because too few valid trials remained after screening eye movement data (see section “Data reduction”). The final sample thus consisted of *N* = 9 participants (three male, six female; average age 25.8 ± 2.6 years). They had normal or corrected-to-normal vision, were right-handed as confirmed by the Edinburgh Handedness Inventory (EHI; mean laterality quotient 73 ± 22; Oldfield 1971) and had no history of known visuo-motor and cognitive deficits. Participants received monetary compensation or course credits for their participation. All experimental procedures were conducted in accordance with the 2008 Declaration of Helsinki and were approved by the local ethical committee.

### Apparatus and stimuli

Subjects were seated at a table on a height-adjustable chair. A personalized bite bar was used to prevent head movements during the experiment, while a head-mounted EyeLink II (SR-Research, Osgoode, ON, Canada) eye tracker recorded movements of the subject’s right eye at a sampling rate of 1 kHz to ensure compliance with the fixation instructions. On the table, a 19-inch touch screen panel (Magic Touch 2.0, Keytec Inc; Garland, TX, USA) was mounted vertically at a distance of 47 cm from the subject’s eyes and recorded reach endpoints at a resolution of 1,920 by 1,080 pixels. Reach to touch movements were performed with the right hand from a home button mounted centrally in front of the participant to the touch screen. Successful touches were confirmed by a beep signal. The touch screen panel and eye tracker were calibrated before the start of each testing session to ensure reliable measurement. Visual stimuli were displayed using seven bi-color (red/green) light-emitting diodes (LEDs) that were controlled via the computer’s parallel port. The LEDs were mounted on an adjustable bar behind the transparent touch screen panel and were spaced five visual degrees (4.11 cm) apart. Red LEDs indicated visual targets which the participants had to fixate and remember, while the green LEDs served as fixation lights. During the experiment, the LEDs were dimmed to prevent illumination of the workspace or the subject’s hand. All stimuli were controlled using Presentation software (Neurobehavioral Systems, Albany, CA, USA). The entire experiment took place in total darkness with the exception of the stimulus LEDs. Additionally, a halogen desk lamp was automatically switched on by the computer between trials to prevent dark adaptation.

### Experimental task

To start the experiment, subjects had to press and hold down a home button mounted on the table. A trial began with the presentation of two red target LEDs in series (T1/T2), each illuminated for 1 s (Fig. 1a, I, II). Targets were always spaced 10° apart and could appear in the following configurations: 10° left/center, 5° left/5° right and center/10° right (see also Fig. 1b for a summary of possible target and fixation positions). In the previous study, a target distance of 5° had been used, which led to predicted error patterns that were close together and therefore hard to distinguish (Thompson and Henriques 2010). Therefore, targets in the present experiment were spaced 10° apart to increase overall reach amplitudes and to better distinguish between egocentric and allocentric contributions. The presentation order of the two target positions was randomized within each condition (50 % left–right and 50 % right–left). Subjects were instructed to fixate each target while it was visible. As the earlier study found no difference between sequential and simultaneous target presentation, we only presented targets sequentially (Thompson and Henriques 2010). Immediately after target presentation or after a delay of 5 or 12 s (Fig. 1a, III), the first green fixation LED (F1) was illuminated for 1 s (Fig. 1a, IV). Subjects were asked to shift gaze to the fixation LED as soon as it was lit and hold fixation at this location during the subsequent reach. When the LED was turned off, subjects lifted their right index finger from the home position and touched the screen where they remembered having seen the first target (Fig. 1a, V). As soon as the reach was registered by the touch screen, a second fixation was displayed for 1 s (F2; Fig. 1a, VI). Again, subjects had to fixate at the new location and hold their gaze here until the end of the second reach. When the LED turned off, they reached to the remembered location of the second target without going back to the home position (Fig. 1a, VII). After this second reach, subjects returned their right index finger to the home position where they pressed and held the button down. Before the next trial started, the desk lamp was switched on for 2 s.

In addition to the experimental condition, we included a gaze-free control condition to estimate subjects’ individual biases of replicating the constant 10° distance between both targets. To do so, they reached for both targets in sequence without being instructed to fixate on a specific location. Therefore, no fixation stimuli were displayed in the control task, which was otherwise identical to the experimental condition.

To be able to modulate gaze position relative to the 10° target array and thus detect potential gaze-dependent variation in reach endpoints, the three possible target array positions (−10°/0°, −5°/5° and 0°/10°) were combined with the fixation positions displayed in Fig. 1b, resulting in gaze displacements of 10° and 15° left and right of the targets. Assuming gaze-dependent coding of the first and second reach targets, this stimulus configuration yields different predictions of both reach endpoints depending on the relative positions of the target and fixation locations. For example, in combination 1 (15° target–fixation distance, Fig. 1b), we would expect both reach endpoints to fall outside the target array (red line) and therefore a larger distance between reach endpoints than the 10° inter-target distance (i.e., larger relative reach errors). In combination 7 and 8 (10° target–fixation distance, Fig. 1b), we would expect both reach endpoints to fall inside the target array, and thus, the distance between reach endpoints should be smaller than 10° (i.e., smaller relative reach errors). This led to six possible combinations with 15° of gaze deviation, where predicted distances between reach endpoints would be larger than the inter-target distance (Fig. 1b: 1–6), but only two with 10° of gaze deviation, where the predicted distances between reach endpoints would be smaller than the inter-target distance (Fig. 1b: 7–8). To balance the number of trials for smaller and larger egocentric predicted distances between reach endpoints, the 10° combinations (combinations 7 and 8 in Fig. 1b) were presented three times in each repetition. In total, the experimental condition consisted of 432 trials for each subject (12 adjusted combinations × 3 delays × 12 repetitions), which were run as three separate sessions of 144 trials each. The control condition was run as a separate session, and the order of control and baseline conditions was counterbalanced across subjects. After each session, a calibration procedure was executed in which each participant had to fixate and touch all possible target locations in sequence, while the halogen lamp was lit. This served as a baseline for each subject’s individual pointing bias and to spatially align eye tracking and touch screen data for offline analysis.

### Data reduction

Touch screen and eye-tracking data were exported into a custom GUI application written in MATLAB (The MathWorks, Natick, MA, USA) where all trials could be viewed across time and selected for analysis or exclusion. Trials were excluded if gaze deviated by more than 2.5° from the instructed target or fixation position, subjects lifted their hand from the home button too early or touched the screen while the second fixation LED was still illuminated. Trials with data recording errors were also removed. Subjects were not included in the final analysis if more than 60 % of trials were removed due to these criteria, which led to the exclusion of one participant. Data were then corrected for outliers by removing all trials with first or second reach errors outside the range of their mean ±3 standard deviations. Across all subjects, 15 % of total trials were excluded from analysis due to the above criteria.

### Statistical analysis

Statistical analyses were performed in MATLAB and R (R Core Team 2013) and were evaluated at an alpha level of 0.05 unless specified otherwise. Post hoc *t* tests were corrected for multiple comparisons using the Bonferroni–Holm method of adjustment. Where Mauchly’s test indicated a violation of the sphericity assumption, Greenhouse–Geisser corrected estimates were used (indicated as *p*_GG_). Error bars in all figures indicate corrected within-subjects standard errors (SE_within_; cf. Cousineau 2005; Morey 2008).

Gaze deviations relative to target were defined as the difference between target locations and the respective fixation location in visual degrees, i.e., a target at +5° combined with a fixation at −10° result in a gaze-relative-to-target distance of +15°. Reach endpoint errors were similarly defined as the angular difference between each actual target location and the corresponding reach endpoint position.

A 3 × 4 repeated measures analysis of variance (RM-ANOVA) with factors delay (0, 5, 12 s) × gaze position (−15°, −10°, 10°, 15°) was used to assess effects of gaze deviation and delay on first and second reach errors. Identical analyses were conducted on variable reach errors for both reach movements, which were defined as standard deviations of horizontal reach endpoint errors.

To quantify the relative contributions of constant allocentric and gaze-dependent egocentric information, we defined reach amplitudes in the second reach as the unsigned distance between first and second reach endpoints in visual degrees. We then calculated the predicted second reach amplitudes for both predictions shown in Fig. 1d. For the egocentric prediction (orange dashed hand), predicted reach errors in the second reach were based on the mean reach errors measured in first reaches at corresponding gaze deviations. For example, the predicted second reach error for a corresponding second fixation of −15° was calculated by averaging endpoint errors in all first reaches where the corresponding gaze deviation was also −15°. Predicted egocentric reach amplitudes of the second reach were then calculated as the unsigned distance between the actual first and the predicted second reaches. For the allocentric prediction of reach amplitudes in the second reach (blue dotted hand), we used participants’ mean perceived distance between the targets, as found in the gaze-free baseline condition (mean distance 12.7° ± 1.5°; see also Fig. 1c). Because data for ±15° of gaze deviation relative to target were made up of six target/fixation combinations, while only two combinations with three times the number of repetitions were used for ±10° of gaze deviation (see also section “Experimental task”; Fig. 1b), the ±10° combinations were tripled in order to balance the number of trials for the egocentric, gaze-centered prediction which should have yielded larger second reach amplitudes (Fig. 1b: 1–6) with those that should have yielded smaller reach amplitudes (Fig. 1b: 7–8) compared with the constant inter-target distance. This was done to ensure that regression analyses yielded correct slopes and were not dominated by the larger number of combinations where we expected reach amplitudes to be larger than the constant relative distance between both targets, therefore resulting in an underestimation of reach error variation with gaze deviation.

If the second target location was only coded using gaze-dependent egocentric information, we would expect the actual amplitudes to vary with gaze in a pattern similar to the predicted amplitudes. If only allocentric information was used to code the second target location, predicted amplitudes should be relatively constant and similar to the perceived allocentric distance between both targets (12.7°). To detect delay-dependent differences in the relative contributions of egocentric and allocentric information, 12 (adjusted target–fixation combinations) × 3 (delays) × 2 (predictions: egocentric vs. allocentric) RM-ANOVA was performed on the unsigned differences between predicted and actual reach amplitudes to assess the fit of each prediction. To further qualify the relative contribution of egocentric and allocentric information on reach amplitudes, we conducted linear regression analysis on egocentrically (gaze-dependent) predicted and actual reach amplitudes for each subject, split by delay conditions. A regression slope of one in this type of analysis would indicate complete reliance on egocentric information, as the actual amplitudes would vary with gaze in the same way as the predictions, while a slope of zero would indicate no relation between the gaze-dependent prediction and the actual reach amplitudes, indicating stronger reliance on the allocentric inter-target distance.

## Results

We investigated whether the contribution of egocentric, gaze-dependent and allocentric reference frames used to represent targets for sequential reach movements depends on a delay between target presentation and reaching. For the first reach in a sequence of two memory-guided reach movements, we predicted gaze-dependent endpoint errors similar to previous studies (Fiehler et al. 2011; Henriques et al. 1998; Schütz et al. 2013). Furthermore, we predicted reach amplitudes for the second reach based either on an egocentric, gaze-dependent reference frame similar to the first reach or on allocentric information based on the constant distance between the first and the second target. To this end, we compared these predictions with the actually measured reach amplitudes.

**Fig. 2:**
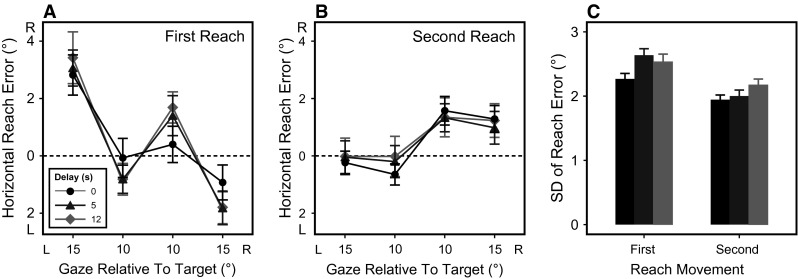
**a, b** Horizontal reach endpoint errors for the first (**a**) and second (**b**) reach movement in visual degrees, plotted as a function of current gaze position relative to target for all delays (*separate lines*). **c** Variable errors (standard deviations of reach endpoint errors) for the three delay conditions in the first and second reach movement (coloring of delays identical to panels **a** and **b**). *Error bars* denote ±1 within-subjects standard error

When analyzing endpoint errors in the first reach, we found a significant pattern of gaze-dependent errors in all delay conditions (*F*_(3, 24)_ = 9.7, *p* < 0.001, $\eta_{G}^{2}$ = 0.42; see also Fig. 2a). Overall, subjects overshot the target toward the right when fixating leftward and vice versa, which is consistent with earlier studies on single-target reaching (Fiehler et al. 2011; Henriques et al. 1998; Schütz et al. 2013).

The gaze-dependent pattern of first reach errors was also influenced by the amount of delay (*F*_(6, 48)_ = 3.6, *p* = 0.004, $\eta_{G}^{2}$ = 0.03; cf. Fig. 2a). When we compared the different delays for each gaze deviation relative to target using Bonferroni–Holm corrected *t* tests, delay and non-delay conditions only differed when gaze was 10° right of the target, showing a trend of undershooting the target when reaching was delayed (0 s vs. 5 s: *p* = 0.087; 0 s vs. 12 s: *p* = 0.087). At all other gaze deviations, the gaze-dependent pattern of reach errors was not significantly different between delays (all *p* > 0.27).

For the second reach movement (shown in Fig. 2b), there was a trend toward reach errors that varied with gaze relative to target (*F*_(3, 24)_ = 2.4, *p* = 0.089), but in contrast to the first reach error, errors were shifted in the direction of the gaze shift (i.e., subjects undershot the target relative to gaze). There was no indication that second reach endpoint errors were influenced by delay (*F*_(6, 48)_ = 0.6, *p* = 0.72).

Figure 2c depicts variable errors for the first and second reach movement, defined as the standard deviations of horizontal reach endpoint errors. Overall, variable errors were smaller in the second than in the first reach movement (*F*_(1, 8)_ = 29.8, *p* < 0.001, $\eta_{G}^{2}$ = 0.15). Additionally, there was a general increase in variability with longer delays across both movements (*F*_(2, 16)_ = 6.3, *p* < 0.001, $\eta_{G}^{2}$ = 0.04).

In order to calculate predicted allocentric reach amplitudes of the second reach for further analysis, we first identified each participant’s individual bias of the fixed distance between the two targets using reach endpoints from the gaze-free baseline condition. Distance biases are shown in Fig. 1c. All subjects overestimated the 10° target distance, yielding reach amplitudes between 10.7° and 15.0°, with a mean amplitude (perceived allocentric target distance) of 12.7° ± 1.5°.

Target and fixation combinations were chosen such that the egocentric predictions fluctuated systematically and substantially, while the amplitude predicted by allocentric information was constant at 12.7°. If subjects used mainly an egocentric representation of the second reach target, we expected gaze-dependent variation in the actual reach amplitudes. However, if they predominantly used the constant target distance (physical: 10°; perceived: 12.7°) as a reference, actual amplitudes of the second reach should fall close to the allocentric prediction with little variation. RM-ANOVA on the unsigned differences between each prediction and the actual amplitudes indicated that reach amplitudes to the second target were generally influenced by current gaze position, as they differed significantly between different target/fixation combinations (*F*_(11, 88)_ = 3.5, *p* < 0.001, $\eta_{G}^{2}$ = 0.10). This modulation across the stimulus combinations shown in Fig. 1b differed between the egocentric and allocentric predictions (*F*_(11, 88)_ = 5.4, *p* < 0.001, $\eta_{G}^{2}$ = 0.10). However, no significant influence of delay on reach amplitudes corresponding to either prediction was found in this analysis.

**Fig. 3:**
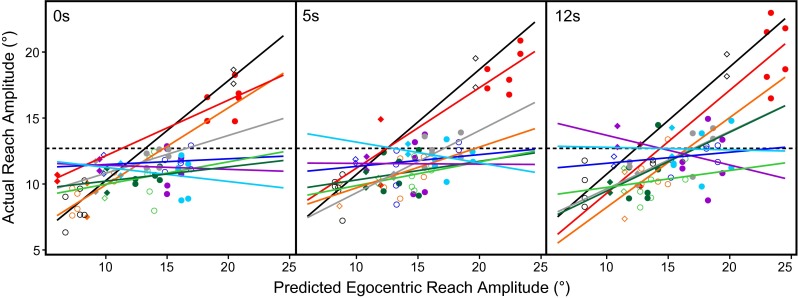
Actual reach amplitudes plotted as a function of reach amplitudes predicted based on gaze-dependent egocentric information, displayed for each subject at all three levels of delay. *Circles* represent combinations with gaze at ±15° (i.e., large expected reach amplitudes). *Diamonds* show combinations with gaze at ±10° (smaller expected reach amplitudes). *Lines* depict *least-squares* regression fit for each subject

To further quantify the relative contribution of gaze-dependent information, linear regression analysis was performed on actual and predicted reach amplitudes of the second reach. Figure 3 plots each subject’s actual reach amplitude per target/fixation combination against the corresponding gaze-dependent prediction, displayed separately for each delay. Thus, if participants relied completely on egocentric gaze information in coding the second reach, the resulting regression slopes should approach unity. A complete reliance on allocentric information from the constant target distance, on the other hand, should yield regression slopes close to zero. Table 1 lists detailed regression slopes for each subject and delay, as well as *t* test results comparing each slope with zero and one. Across delays, the majority of subjects yielded slopes that were significantly different from one, while only three out of nine subjects showed slopes significantly different from zero.

**Table 1:** Regression slopes and statistics of predicted versus actual reach amplitudes for each subject, split by delay

Subject	Delay	Slope	*R* ^2^	Slope = 0?		Slope = 1?	
				*t*	*p*	*t*	*p*
1	0	0.42	0.93	6.28	0.001**	−8.64	0.000***
	5	0.61	0.72	4.15	0.006**	−2.62	0.039*
	12	0.78	0.82	4.19	0.006**	−1.19	0.278
2	0	0.58	0.26	2.06	0.085	−1.49	0.187
	5	0.31	0.36	1.43	0.204	−3.55	0.012*
	12	0.68	0.29	1.60	0.161	−0.88	0.413
3	0	−0.03	0.01	−0.14	0.890	−5.41	0.002**
	5	−0.01	0.00	0.00	0.997	−2.68	0.044*
	12	−0.23	0.22	−0.88	0.410	−5.35	0.002**
4	0	0.17	0.06	0.45	0.669	−2.37	0.055
	5	0.18	0.20	1.05	0.334	−4.08	0.006**
	12	0.13	0.05	1.13	0.301	−3.25	0.017*
5	0	0.11	0.08	0.95	0.381	−6.50	0.001**
	5	0.14	0.04	0.33	0.752	−2.75	0.033*
	12	0.45	0.22	1.23	0.265	−1.11	0.311
6	0	0.04	0.04	0.56	0.598	−9.87	0.000***
	5	0.09	0.05	0.95	0.381	−4.44	0.004**
	12	0.08	0.20	1.26	0.255	−10.27	0.000***
7	0	−0.11	0.07	−0.54	0.606	−5.27	0.002**
	5	−0.16	0.13	−0.53	0.618	−5.49	0.002**
	12	−0.02	0.00	0.31	0.769	−2.69	0.036*
8	0	0.76	0.96	9.46	0.000***	−3.03	0.023*
	5	0.80	0.94	8.02	0.000***	−2.08	0.083
	12	0.81	0.91	5.89	0.001**	−1.87	0.111
9	0	0.29	0.41	2.18	0.073	−5.39	0.002**
	5	0.47	0.67	2.48	0.048*	−3.30	0.016*
	12	0.44	0.59	2.52	0.045*	−4.07	0.007**
Mean	0	0.25	0.31				
	5	0.27	0.35				
	12	0.35	0.37				

Right side of the table shows slope *t* test results for comparison with a slope of 0 (no gaze-dependent modulation, i.e., equal to purely allocentric coding) as well as a slope of 1 (perfect gaze-dependent prediction, i.e., equal to purely egocentric coding)

* *p* < 0.05; ** *p* < 0.01; *** *p* < 0.001

**Fig. 4:**
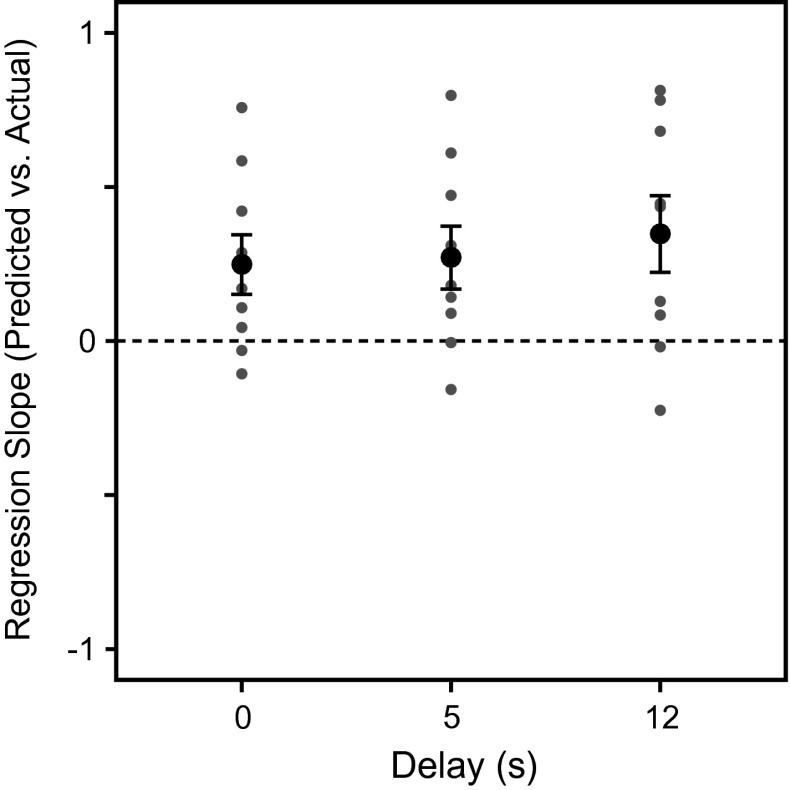
Comparison of mean regression slopes from Fig. 3 for each delay condition. *Small dots* indicate individual subject means. *Large dots* represent overall mean slope and corresponding standard errors. A slope of 1 would mean purely egocentric coding of the second target location. A slope of 0 would indicate complete reliance on constant allocentric information

To facilitate visual comparison of slopes across delays, Fig. 4 depicts the regression slopes from Fig. 3 for each subject as well as the grand mean for each amount of delay. Average slopes did not differ significantly between delays (*F*_(2, 16)_ = 1.7, *p* = 0.21), indicating that delay did not influence the relative contribution of gaze-dependent information on actual reach amplitudes.

## Discussion

The present study investigated the reference frames used to represent visual targets for sequential-reaching movements, and whether these reference frames are influenced by a delay between target viewing and reaching. In particular, we were interested in the spatial coding scheme of the second reach target which could be either egocentric or allocentric, i.e., relative to gaze or relative to the position of the first reach target, respectively. We found evidence for a combined use of egocentric and allocentric representations of second reach targets, but no indication that the relative contribution of both types of representation changes with memory delay.

### First reach movement

The first target in the sequence was presented without any other visual cues. Therefore, we expected gaze-dependent reach errors for the first target similar to other studies that used a single reach goal (Dessing et al. 2012; Fiehler et al. 2011; Henriques et al. 1998; Medendorp and Crawford 2002). Indeed, we found that when subjects reached to the first target, they generally overshot the target to the right when fixating left and vice versa, which agrees with our prediction and is consistent with the previous study on sequential reaching by Thompson and Henriques (2010). The similarities of this error pattern to previous findings suggest that subjects utilized primarily egocentric, gaze-dependent target representations when coding the first reach.

### Second reach movement

To compare the relative contributions of both egocentric, gaze-dependent and allocentric information in the second reach movement where its constant distance to the first target provided a stable visual cue, we calculated actual and predicted reach amplitudes in the second reach. The egocentric prediction was based on the first reach error pattern described above, while the allocentric prediction was based on the constant distance between the first and second reach targets. Therefore, the amount of gaze-dependent modulation in the actual reach amplitudes of the second reach is directly related to the contribution of gaze-dependent egocentric information: If subjects used mainly egocentric information, the actual second reach amplitudes should vary with gaze similar to the first reach endpoints; if they used allocentric information, such a systematic gaze-dependent variation should not occur.

We found gaze-dependent modulation of the actual second reach amplitudes, which suggests that gaze direction (at least partially) contributed to reach target encoding. Moreover, the actual reach amplitudes were distinct from both the egocentric and the allocentric prediction, indicating that they lay between both predictions. Similar results have been published in studies on reaching to single targets where additional landmarks were present and interpreted as a combination of egocentric and allocentric information in goal-directed action (Byrne et al. 2010; Byrne and Crawford 2010; Byrne and Henriques 2012; Schütz et al. 2013). How these representations are combined is still a topic of investigation, with some reports arguing for optimal integration of both sources (Byrne and Crawford 2010) and others for a cue switching strategy (Byrne and Henriques 2012).

To better quantify the influence of gaze direction on the second reach, we calculated regression slopes of predicted versus actual reach amplitudes. A dominant use of an egocentric, gaze-dependent reference frame would result in a slope around one, while a dominant use of an allocentric reference frame would result in a slope around zero. The majority of individual subjects’ slopes were significantly different from one but not from zero, suggesting that egocentric, gaze-centered information was not used predominantly in coding the second reach targets. With a mean slope of about 0.3 across all delays, allocentric information had a strong influence, possibly more so than in the previous sequential-reaching study by Thompson and Henriques (2010). These findings are also in line with our previous work, where we found reduced gaze-dependent reaching errors for single-reaching movements when landmarks were present (Schütz et al. 2013). However, the contribution of an egocentric, gaze-dependent reference frame was clearly smaller in the present experiment, since the resulting error pattern in our previous work was significantly influenced by gaze direction, whereas here, we found only a statistical trend. Hence, allocentric information seems to have a stronger influence on reaching movements in the present study compared with our previous one (Schütz et al. 2013). A possible explanation of this difference is given by recent findings from our group which demonstrated that allocentric cues are only effectively used for reaching if they are task-relevant, i.e., potential reach targets (Fiehler et al. 2014; Klinghammer et al. 2014). Both the present study and the earlier report by Thompson and Henriques (2010) found a strong influence of allocentric coding in the sequential reach paradigm, where the distance between targets provided stable allocentric information, while at the same time, the first target served as an intermediate reach goal for the second target. Therefore, it is plausible that the brain preferentially integrates spatial information about visual cues into the movement plan if they are relevant for an upcoming movement.

Taken together, we found evidence for a combined use of egocentric, gaze-dependent information about the second target location and allocentric information from the fixed distance between both targets, with a stronger contribution of the latter. This agrees well with the current literature (Byrne and Crawford 2010; Byrne and Henriques 2012; Fiehler et al. 2014) and reports from both single-reaching (Chen et al. 2011; Schütz et al. 2013) and sequential-reaching tasks (Thompson and Henriques 2010).

It should be noted that the paradigm we used can only distinguish between gaze-dependent and gaze-independent frames of reference, similar to other studies that have used manipulations of gaze position while the head was fixed to investigate action goal coding (Henriques et al. 1998; Medendorp and Crawford 2002; Fiehler et al. 2011; Khan et al. 2005a, b; Schütz et al. 2013). Therefore, we cannot rule out the possibility that gaze-independent reference frames other than an allocentric frame, such as an egocentric head- or body-centered one, could have been used to code both target locations, resulting in similar reach amplitudes. Nevertheless, the brain has to have stored the relative locations of both targets and used this information in planning the reach movement in addition to a simple updating of target locations with each gaze shift, as the gaze-dependent component of the reach amplitudes was very small compared with the consistent distance information. Recently, Chen et al. (2011) have argued that humans tend to convert allocentric cues into an egocentric representation as soon as possible. If this is the case and the egocentric representations of both targets and the inter-target distance then decayed equally over time (Hesse and Franz 2009, 2010), we would expect no change in the relative contribution of both information sources but a generally increasing variability of reach endpoints with delay. Our results agree well with this assumption, and we therefore cannot rule out the possibility of landmark information being converted into a gaze-independent egocentric representation before the reach. Moreover, in the current experiment, the 10° distance between targets remained the same throughout the study, and thus, there was no systematic manipulation of allocentric information comparable with the egocentric gaze shifts.

### Effect of delay on spatial coding

We found no evidence that the amount of delay between viewing both targets and executing the corresponding reach movements influenced the relative contribution of egocentric and allocentric information. Additionally, regression slopes quantifying the relative contribution of gaze-dependent egocentric information did not vary with the amount of delay. It has been argued that immediate action relies on an egocentric representation of spatial target locations, and that our visuo-motor system switches to a gaze-independent allocentric code as soon as the action is delayed (Goodale and Milner 1992; Hu and Goodale 2000; Westwood et al. 2000; Westwood and Goodale 2003). Following this assumption, we would have expected a steep slope (i.e., not different from unity) in the immediate reaching condition, which should become indistinguishable from zero or at least be significantly smaller in the delayed reaching condition. In contrast, we found a strong reliance on allocentric information already for immediate reaching but no further increase in allocentric contribution with delay. In previous work, we have already shown that the presence of visual landmarks similarly influences both immediate as well as delayed actions, leading to improved accuracy and precision of reaching movements (Schütz et al. 2013). The present experiment suggests that the contribution of allocentric information to reaching is enhanced when it is task-relevant and that this contribution does not increase with memory delay. Our results therefore do not support the hypothesis of separate processing systems for immediate and memory-guided action, rather they emphasize that both types of representation work closely together in goal-directed movements irrespective of delay.

Variable errors increased with longer delays both in the first and second reach of the current experiment, which is in line with previous reports (Obhi and Goodale 2005; Fiehler et al. 2011). At least for the first reach where no allocentric information was available, this agrees well with the assumption that egocentric information decays over time and therefore becomes noisier (Chen et al. 2011; Hesse and Franz 2009, 2010). In some cases where reach endpoint variability was assessed when allocentric landmarks were present, no increase in variable errors has been reported (Chen et al. 2011; Schütz et al. 2013). However, in these studies, the landmarks were static visual cues that were either present during the subsequent reach (Schütz et al. 2013) or at least presented again immediately before reach onset (Chen et al. 2011). Here, the first target was only presented for a short time during the first part of the trial and then had to be kept in memory until the reach. The observed attenuation of increased variable errors with delay may therefore depend on the time frame or reliability of the first target, which was shown only briefly in the present experiment and therefore might not have contributed much to a reduction in variability.

## Conclusions

Taken together, the presented findings indicate that when executing goal-directed reach movements, the brain integrates egocentric gaze-dependent with allocentric spatial information. However, even when the allocentric cues were directly relevant to the reach plan by serving as intermediate reach targets in a sequence, their influence on the representation used to execute the movement sequence did not change when movements were delayed. To conclude, our results argue against a complete switch from egocentric to allocentric coding of reach targets in sequential reaching, even when movements are delayed, but suggest a combined use of egocentric and allocentric information irrespective of delay.
